# Applying Rasch analysis to the SF-36 physical function scale: effect of dependent items

**DOI:** 10.1186/1745-6215-12-S1-A75

**Published:** 2011-12-13

**Authors:** Mike Horton, Alan Tennant

**Affiliations:** 1 Psychometric Laboratory for Health Sciences, Department of Rehabilitation Medicine, The University of Leeds, UK

## 

Medical outcome studies often use Patient Reported Outcomes (PROs), and these often take the form of administered or self-completed questionnaires.

Often, the responses from these scales are simply added up as a total, and this total score is utilised for outcome purposes. However, for this approach to be valid, there are a number of underlying assumptions that are being made. One of these assumptions is that of Local Independence, which comprises two aspects; the items making up the scale should all be unidimensional (trait dependency) and, the response to an item should not directly influence the response to another item within the set (response dependency) [1].

These assumptions apply to the Rasch model (and other IRT models). Thus the process of Rasch analysis provides a means to test these assumptions, along with other key properties such as invariance. Where data satisfy these assumptions, and fit to the model, an interval scale transformation becomes available.

A secondary analysis was performed on data from the Physical Function scale (PF-10) of the SF-36 [2] to demonstrate the application of Rasch Analysis and to investigate the influence of response dependency within the dataset.

Initial results showed evidence of a lack of unidimensionality (t-tests = 8.39%; lower bound CI = 5.9%), along with apparent response dependencies between the three walking items of the PF-10 (walking 100 yards, walking half a mile & walking more than a mile), the two climbing stairs items of the PF-10 (climbing one flight of stairs & climbing several flights of stairs) and a dependency between the ‘moderate activities’ item and the ‘lifting or carrying groceries’ item. When adjustments had been made for the response dependency within the item set, it then appeared to be unidimensional (t-tests = 2.1%).

Also to be noted is the artificial inflation of the reliability index due to the dependency within the item set. After dependency adjustments had been made, the reliability index (PSI) dropped from 0.851 (initially) to 0.789 (Cronbach’s alpha dropped from 0.91 to 0.80).

In conclusion, response dependency can inflate reliability, and lead to spurious (dependency) factors. It has also been shown to influence both item and person parameters in IRT analysis. Rasch analysis provides a framework to assess, as well as adjust for, response dependency within an item set.
